# The impact of diagnosis-related groups on medical costs, service efficiency, and healthcare quality in Meishan, China: An interrupted time series analysis

**DOI:** 10.1371/journal.pone.0325041

**Published:** 2025-05-22

**Authors:** Jinyuan Li, Meihua Peng, Shihu Deng, Jingwen Wu, Wangsu Zhou

**Affiliations:** 1 College of Management, Chengdu University of Traditional Chinese Medicine, Chengdu, Sichuan Province, China; 2 Meishan Healthcare Security Administration, Meishan, Sichuan Province, China; Centre Hospitalier de Troyes, FRANCE

## Abstract

**Background:**

In January 2019, the Diagnosis-Related Groups (DRG) payment system was introduced in Meishan, China. Using the medical insurance records from 2017 to 2022, we evaluated the impact of the DRG system on medical costs, service efficiency and healthcare quality.

**Methods:**

The sample was divided into three periods: Before DRG reform (2017–2018), the first period of DRG reform (2019–2020), and the second period of DRG reform (2021–2022). We employed an Interrupted Time Series (ITS) model to analyze the monthly changes in total hospital costs, patient cost-sharing, patient sharing ratio, length of stay, and 30-day readmission rate during both periods of DRG reform.

**Results:**

In the first period of DRG reform, total hospital costs decreased by 1.23% per month (95% CI, 0.88%-1.59%), patient cost-sharing decreased by 1.46% per month (95% CI, 1.09%-1.83%), patient sharing ratio decreased by 0.23% per month (95% CI, 0.06%-0.40%), and length of stay decreased by 0.56% per month (95% CI, 0.27%-0.84%). The monthly change in 30-day readmission rate was not statistically significant (-0.11%, 95% CI, -0.73%-0.50%). In the second period of DRG reform, all monthly changes were not statistically significant.

**Conclusions:**

This study assessed the impact of the DRG payment system on medical costs and service efficiency. The results showed that DRG reduced total hospital costs, patient cost-sharing, patient sharing ratio, and length of stay, but did not significantly affect the rising 30-day readmission rates. Over time, the impact of DRG on cost control and service efficiency stabilized. However, unintended hospital behaviors may have emerged, warranting further investigation. The findings suggest that policymakers should strengthen clinical practice regulation, improve the DRG payment system, and continuously monitor healthcare quality trends.

## Introduction

Over the past few decades, many countries have experienced rapid increases in healthcare costs, primarily driven by the overuse of medical services under the Fee-For-Service (FFS) model [1,2]. In response, many countries have gradually implemented the Diagnosis-Related Groups (DRG) payment system to control escalating healthcare expenses while improving hospital efficiency and service transparency [3]. As a form of case-based payment or activity-based funding, DRG groups patients based on their characteristics, allowing healthcare payers to provide fixed compensation to medical institutions and facilitating comparisons between them [4].

Developed countries such as those in Europe and the United States have accumulated substantial experience in the implementation of DRG. Some studies have concluded that the introduction of the DRG payment system may result in a range of anticipated and unanticipated changes in healthcare costs and service efficiency [5,6]. Theoretically, DRG systems are expected to enhance hospital efficiency by providing fixed payments that incentivize cost-effective practices, as hospitals have a strong financial motivation to reduce the use of medical resources and select the least costly inputs. Given these incentives, the intensity and duration of care for patients are anticipated to decrease, leading to shorter lengths of stay and potentially reducing overall healthcare costs. However, empirical evidence is mixed; while some studies report significant reductions in hospital costs [7–9], others have found that DRG reforms have not consistently resulted in cost savings [10]. Moreover, the reduction in the length of stay, although effective in controlling costs, has raised concerns about the quality of care, with some studies suggesting an increase in readmission rates as a consequence of shorter hospital stays [11].

As one of the fastest-growing developing countries, China’s healthcare costs have surged dramatically due to inefficient use of medical services under the FFS model [12]. In response, many Chinese cities have adopted healthcare reforms, with DRG serving as a representative model [13–16]. In 2018, following an initiative by the Meishan Municipal Government, the city was approved by the Sichuan Provincial Human Resources and Social Security Department as a pilot city for the DRG-based payment reform. In January 2019, 29 Grade II and Grade III hospitals were selected to participate in the DRG reform. The inclusion criteria for these hospitals were based on their capacity to meet the technical and administrative requirements necessary for DRG implementation, including adequate hospital scale, information infrastructure, coding ability, and overall management capacity. Primary care institutions were excluded at this stage due to limited capabilities. This government-imposed mandate effectively created an exogenous shock, as hospitals had no discretion in deciding whether to participate, thus providing a clear and abrupt policy change for evaluation.

As the DRG reform progressed, hospitals gradually adapted to the new payment system, refining their cost control strategies. Given the potential for these reforms to influence hospital efficiency and costs in both expected and unforeseen ways, we introduce a second period of DRG reform beginning in January 2021 to further evaluate the sustainability of the DRG system’s impact. However, it is important to clarify that this second period does not correspond to any formal policy change, but rather serves as a continuation of observation following the initial implementation.

Compared to countries in Europe and America, China’s DRG reform is relatively recent. Given the disparities in healthcare service capacity and resources, the reform experiences of developed countries may have limited applicability for guiding the implementation of DRG in developing countries.

To the best of our knowledge, this study represents the longest evaluation of the DRG system in Meishan, China, and serves as a valuable supplement to existing research. We provide a more comprehensive analysis of trends following the introduction of DRG, addressing the limitations of previous studies with shorter evaluation periods, and offering policy recommendations to optimize the DRG payment system.

## Methods

### Data source

Our dataset, obtained from the Meishan Healthcare Security Administration, includes 1,617,608 medical insurance records from 29 pilot hospitals. It includes detailed information on patient characteristics (such as age, gender, and insurance type), hospitalization services (including admission and discharge dates and whether readmission occurred within 30 days), hospital level (Grade II and Grade III), ownership (public and private), diagnoses, costs, and medical insurance reimbursement details.

It should be noted that prior to data collection, the Meishan Healthcare Security Administration anonymized patients’ personal identity information, and the dataset does not contain any identifiable personal information, thus ethical approval is not required. As this study is based on administrative data routinely collected by the Meishan Healthcare Security Administration, patient consent is not required.

### Variables

Our study includes five outcome variables: (1) total hospital costs; (2) patient cost-sharing: In China’s medical insurance system, patient cost-sharing consists of three components: first, expenses outside the scope of basic medical insurance coverage; second, expenses within the coverage scope that require patient co-payment at a certain percentage; and third, out-of-pocket expenses, including deductibles and costs outside the coverage scope; (3) patient sharing ratio; (4) length of stay: used to measure hospital service efficiency; (5) 30-day readmission rate: used to assess changes in healthcare quality. To effectively mitigate the impact of price fluctuations on the research data, this study uses 2017 as the base year and adjusts the cost-related data from 2018 to 2022 to the same price level as 2017, based on the regional Consumer Price Index (CPI). Table 1 summarizes the measurement methods for the outcome variables and control variables.

**Table 1:** Variables name and variable measurement.

	Variables Name	Variable Measurement
Outcome Variables	Total hospital costs	The total hospital costs incurred in patient’s hospitalization
	Patient cost-sharing	Amount of total hospital costs paid by the patient
	Patient sharing ratio	Divided patient cost-sharing by total hospital costs
	Length of stay	Patient discharge date minus patient admission date
	30-day readmission rate	Constructed as a dichotomous variable (0 or 1) at the patient-level
Control Variables	Gender	female = 0, male = 1
	Age	Age of the patient
	Insurance type	Employee Basic Medical Insurance (EBMI) = 0, Resident Basic Medical Insurance (RBMI) = 1
	Charlson Comorbidity Index (CCI)	Measured patient severity based on ICD-10 codes of secondary diagnoses.
	Hospital level	Grade II hospitals = 0, Grade III hospitals = 1
	Hospital ownership	Private hospitals = 0, Public hospitals = 1

### Empirical approach

To assess the impact of the DRG reform implemented in January 2019 and to examine the potential ongoing effects of the policy, we hypothesize the occurrence of a second policy shock in January 2021. Although this second shock is hypothetical and does not reflect any actual policy changes, it enables us to explore the potential long-term effects and further refine our understanding of the DRG reform’s impact. We use the Interrupted Time Series (ITS) model to analyze changes in various outcome variables before DRG reform (January 2017 to December 2018), during the first period of DRG reform (January 2019 to December 2020), and during the second period of DRG reform (January 2021 to December 2022). The model is specified as follows:


$$\begin{array}{c} \text{Yt= }\beta \;\;\text{ 0+ }\;\;\beta \;\;\text{ 1Tt+ }\beta \;\;\text{ 2DRG1t+ }\beta \;\;\text{ 3TtDRG1t+ }\beta \;\;\text{ 4DRG2t+ }\beta \;\;\text{ 5TtDRG2t+}\alpha \text{Xt+}\varepsilon \text{t} \end{array}$$ (1)

where Y_t_ represents the outcome variable for month t. T_t_ is the time elapsed since the beginning of the study (coded 1–72). DRG_1t_ and DRG_2t_ are dummy variables representing pre-intervention (coded 0) and post-intervention (coded 1) periods. T_t_DRG_1t_ and T_t_DRG_2t_ are interaction terms for time and the intervention. X_t_ is a vector of control variables at the year-month level, including age, gender, insurance type, CCI, hospital level, and hospital ownership. We use fourier transform to control for seasonality [17]. β_0_ represents the baseline level of the outcome variable when T_t_ = 0, β_1_ represents the potential trend before the DRG reform, β_2_ represents the immediate change from the initial policy shock, β_3_ the monthly change following the initial policy shock, β_4_ represents the immediate change from the hypothesized second policy shock, and β_5_ represents the monthly change from the hypothesized second policy shock.

Considering that the variables for total hospital costs, patient cost-sharing, patient sharing ratio, and length of stay exhibit skewed distributions, we employ a Generalized Linear Regression (GLM) model with a log link function for estimation [18]. For the count data (30-day readmission rate), we utilize a Negative Binomial Model (NBM) to account for overdispersion.

Meaningful changes in clinical practice under a new payment system take time to capture the delayed effects. Consequently, we conducted a lagged analysis of the initial policy shock, rather than examining immediate changes following the policy implementation. In addition, we performed a value-for-money estimation using the GLM without log link. Given that the DRG reform may affect patients across different age groups in distinct ways [19], we performed a stratified analysis by age. All statistical analyses were carried out using Stata 17.0, with a significance level set at α = 0.05.

## Results

Table 2 presents the sample characteristics and outcome variables of our study, which include 1,617,608 inpatient discharge cases across the three periods.

**Table 2:** Descriptive statistics of patient characteristics and outcome variables before, Phase I and Phase II implementation of DRG.

Variables	Before DRG reform	First period of DRG reform	Second period of DRG reform
Patient characteristics			
Discharge case, No.	511857	556594	549157
Male, No.(%)	239125 (46.72)	262110 (47.09)	262075 (47.72)
Age, mean (SD)	53.80 (24.23)	52.65 (24.35)	54.13 (23.82)
65 years old and older, No.(%)	210611 (41.15)	219214 (39.38)	225899 (41.14)
Insurance type, No.(%)			
EBMI	85766 (16.76)	92220(16.57)	100951(18.38)
RBMI	426091 (83.24)	464374(83.43)	448206(81.62)
CCI, mean (SD)	0.46 (1.02)	0.74 (1.59)	1.00 (1.99)
Hospital level, No.(%)			
Grade II hospitals	250014 (48.84)	270837 (48.66)	243877 (44.41)
Grade III hospitals	261843 (51.16)	285757 (51.34)	305280 (55.59)
Hospital ownership, No.(%)			
Private hospitals	99537 (19.45)	107213 (19.26)	102607 (18.68)
Public hospitals	412320 (80.55)	449381 (80.74)	446550 (81.32)
Outcome Variables, mean (SD)			
Total hospital costs	6852.98 (8051.50)	6902.17 (8254.53)	6736.99 (8580.69)
Patient cost-sharing	3548.32 (4683.70)	3472.32 (3993.80)	3167.88 (4699.32)
Patient sharing ratio (%)	53.39 (13.74)	50.56 (13.56)	47.46 (12.96)
Length of stay	8.68 (6.25)	8.32 (5.75)	8.02 (5.43)
30-day readmission rate (%)	8.66 (0.59)	10.45 (0.89)	11.61 (0.71)

DRG denoted the Diagnosis-Related-Group. The study before the DRG reform was from January 2017 to December 2018; the first period of DRG reform was from January 2019 to December 2020; and the second period of DRG reform was from January 2021 to December 2022. In order to effectively remove the impact of price fluctuations on the study data, this study used 2017 as the base period and adjusted the 2018–2022 cost data to the same price level as 2017 based on the CPI (Consumer Price Index) for the region. EBMI: employee basic medical insurance; RBMI: resident basic medical insurance.

We observed that after the DRG reform, the proportion of male patients increased, while the proportion of elderly patients aged 65 and above initially decreased but then increased. More than 80% of patients were covered by the resident insurance scheme and primarily sought care at public hospitals. Compared to the pre-reform period, total hospital costs increased during the first period after the DRG reform but decreased in the second period. Patient cost-sharing, patient sharing ratio, and length of stay all decreased significantly in both post-reform periods, while the 30-day readmission rate increased noticeably.

**Fig 1:**
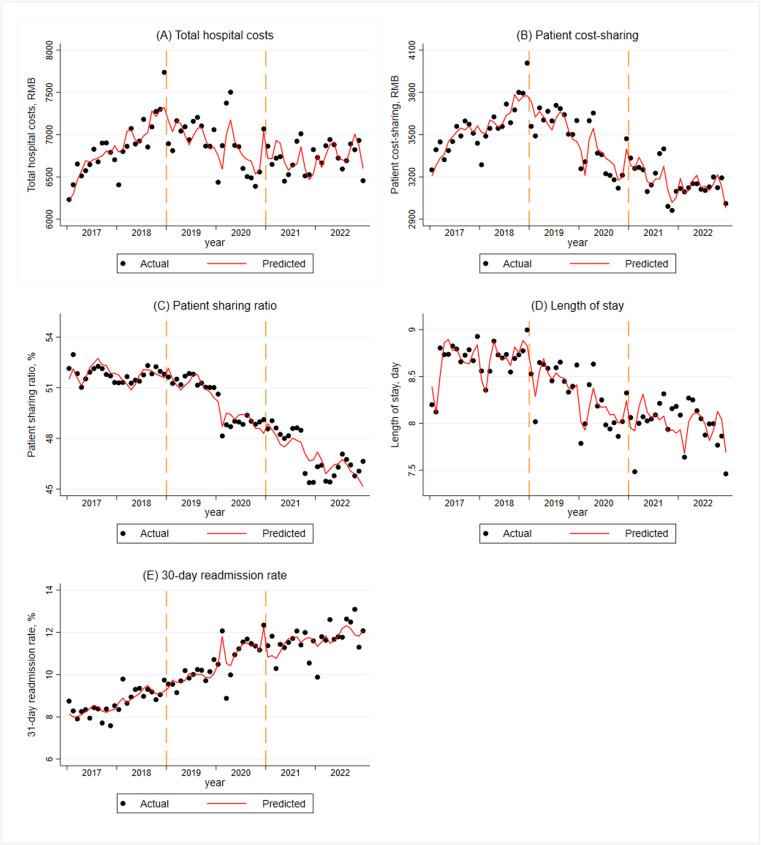
Monthly trends in total hospital costs, patient cost-sharing, patient sharing ratio, length of stay and 30-day readmission rate. Notes: The first dashed line represents the initial policy shock (implementation of the DRG payment reform in January 2019), while the second dashed line indicates the second policy shock (a hypothetical intervention assumed for further observation of outcome changes). The red trend line is derived from segmented regression predictions of the time series model. Outcomes were adjusted for gender, age, insurance type, Charlson Comorbidity Index, hospital level, hospital ownership and seasonality.

Table 3 summarizes the baseline trend, immediate changes and monthly changes of outcome variables. The Fig 1 presents changes in outcome variables during the three periods.

**Table 3:** Interrupted time series (ITS) analyses for total hospital costs, patient cost-sharing, patient sharing ratio, length of stay and 30-day readmission rate of hospitalized patients before and after the DRG.

Outcome Variables	Before DRG reform		First period of DRG reform		Second period of DRG reform	
	Constant (β0) coefficient (95% CI)	Baseline monthly slope change (β1) coefficient (95% CI)	Immediate change (β2) coefficient (95% CI)	Monthly change (β3) coefficient (95% CI)	Immediate change (β2) coefficient (95% CI)	Monthly change (β5) coefficient (95% CI)
Total hospital costs	9.4976^***^	0.0039^***^	0.0002	−0.0123^***^	0.0609^***^	0.0032^*^
	(8.1899–10.8053)	(0.0019–0.0059)	(−0.0429–0.0433)	(−0.0159–−0.0088)	(0.0163–0.1055)	(−0.0004–0.0068)
Patient cost-sharing	8.6851^***^	0.0028^***^	0.0261	−0.0146^***^	0.0790^***^	0.0022
	(7.3181–10.0521)	(0.0007–0.0049)	(−0.0190–0.0712)	(−0.0183–−0.0109)	(0.0323–0.1256)	(−0.0015–0.0060)
Patient sharing ratio	3.7864^***^	−0.0011^**^	0.0258^**^	−0.0023^***^	0.0178	−0.001
	(3.1581–4.4146)	(−0.0020–−0.0001)	(0.0051–0.0466)	(−0.0040–−0.0006)	(−0.0036–0.0392)	(−0.0027–0.0008)
Length of stay	4.1712^***^	0.0002	−0.02	−0.0056^***^	0.0365^**^	0.0018
	(3.1273–5.2150)	(−0.0014–0.0018)	(−0.0545–0.0144)	(−0.0084–−0.0027)	(0.0009–0.0721)	(−0.0011–0.0047)
30-day readmission rate	−2.1359^*^	0.0089^***^	−0.0803^**^	−0.0011	−0.0681^*^	−0.0004
	(−4.4186–0.1467)	(0.0054–0.0124)	(−0.1557–−0.0048)	(−0.0073–0.0050)	(−0.1448–0.0086)	(−0.0066–0.0059)

DRG denoted the Diagnosis-Related-Group; CI the confidence interval. All estimated coefficients were generated from generalized linear regression with log link. The estimated coefficients were directly interpreted as marginal effects. For example, an estimated coefficient of 0.0039 implied a 0.39% monthly increase in total hospital costs before the DRG reform. ITS analyses controlled for gender, age, insurance type, Charlson Comorbidity Index, hospital level, hospital ownership and seasonality.

* p < 0.1,

** p < 0.05,

*** p < 0.01

The monthly trend of total hospital costs significantly increased by 0.39% (95% CI, 0.19%-0.59%) before the DRG reform. In the first period of DRG reform, we found no immediate change in total hospital costs (0.02%, 95% CI, -4.29% to 4.33%), but a monthly decrease of 1.23% (95% CI, 0.88%-1.59%). In the second period, total hospital costs immediately and significantly increased by 6.09% (95% CI, 1.63%-10.55%), with a monthly increase of 0.32% (95% CI, -0.04% to 0.68%), though the result was not statistically significant (Table 3 and Fig 1A).

Patient cost-sharing significantly increased by 0.28% per month (95% CI, 0.07%-0.49%) before the introduction of DRG. In the first period of DRG reform, the immediate change was not significant (2.61%, 95% CI, -1.90% to 7.12%), but a significant monthly decrease of 1.46% (95% CI, 1.09%-1.83%). In the second period, patient cost-sharing showed a significant immediate increase of 7.90% (95% CI, 3.23%-12.56%), but the monthly change was not statistically significant, with an increase of 0.22% (95% CI, -0.15% to 0.60%) (Table 3 and Fig 1B).

The patient sharing ratio decreased significantly by 0.11% per month (95% CI, 0.01%-0.20%) before DRG reform. In the first period of DRG reform, the immediate change was a significant increase of 2.58% (95% CI, 0.51%-4.66%), followed by a significant monthly decrease of 0.23% (95% CI, 0.06%-0.40%). In the second period, both the immediate change (1.78%, 95% CI, -0.36% to 3.92%) and the monthly change (0.10%, 95% CI, -0.27% to 0.08%) in patient sharing ratio were not statistically significant (Table 3 and Fig 1C).

Before the introduction of DRG, we observed no significant monthly change in length of stay (0.02%, 95% CI, -0.14% to 0.18%). In the first period of DRG reform, the immediate change was not significant (-2.0%, 95% CI, -5.45% to 1.44%), but a significant monthly decrease of 0.56% (95% CI, 0.27%-0.84%) was observed. In the second period, the immediate change increased significantly by 3.65% (95% CI, 0.09%-7.21%), while the monthly change (0.18%, 95% CI, -0.11% to 0.47%) was not statistically significant (Table 3 and Fig 1D).

We found that the 30-day readmission rate increased by 0.89% per month (95% CI, 0.43%-1.27%) before the introduction of DRG. In the first period of DRG reform, the immediate change increased significantly by -8.03% (95% CI, -15.57% to -0.48%), but the monthly change was not significant (-0.11%, 95% CI, -0.73% to 0.50%). In the second period, both immediate and monthly changes were decreased, but none of these were statistically significant (Table 3 and Fig 1E).

S1 and S2 Tables present the results of the lagged analysis and age-stratified analysis. We found that, with a 6-month lag, the immediate changes related to costs shifted from an increase to a decrease after the introduction of DRG. The age-stratified analysis revealed that in the first period after the introduction of DRG, the greatest monthly reductions in total hospital costs (1.43%, 95% CI, 1.01%-1.86%) and length of stay (0.83%, 95% CI, 0.52%-1.14%) were observed among patients aged 65 and above. In the second period, the largest monthly increases were observed in total hospital costs (0.63%, 95% CI, 0.27%-1.00%) and length of stay (0.48%, 95% CI, 0.21%-0.75%). S3 Table presents the results of a value-for-money estimation based on ITS analysis. Additionally, the number of inpatients per period, as reported in Table 2, can be used in conjunction with these monthly averages to estimate the overall financial impact of the policy.

## Discussion

This study used ITS to assess the impact of DRG reform on medical costs, service efficiency, and healthcare quality. Currently, in the field of health policy evaluation, the Difference-In-Differences (DID) method seems to be favored by researchers [20]. DID is typically used to estimate the average treatment effect of an intervention and requires meeting the parallel trends assumption [21]. Therefore, ITS serves as a suitable and effective analytical framework in the absence of a control group. It not only constructs counterfactual scenarios by establishing potential trends through time series data [17], but also enables flexible adjustments for underlying trend factors, such as seasonal fluctuations, cyclical trends, and lagged effects, thereby improving the precision of intervention effect estimates [22].

This study found that the monthly trends of total hospital costs, patient cost-sharing, patient sharing ratio, and length of stay declined during the first period of DRG reform. In the second period, total hospital costs, patient cost-sharing, and length of stay showed a nonsignificant upward trend. The two periods after the introduction of DRG slowed the increase in the 30-day readmission rate, though this result was not statistically significant. These findings suggest that improvements in cost and efficiency indicators are associated with the DRG reform, while no significant improvements were observed in quality indicators.

Before the introduction of DRG, the main payment system for medical insurance was FFS, which led to excessive prescriptions and unnecessary tests [23,24]. The first period of DRG reform effectively reduced medical costs and length of stay, a finding consistent with conclusions from other pilot cities in China [25,26] as well as from studies in Europe and America [27–30]. The DRG reform may have stimulated changes in clinical practices, as the fixed payment structure created marginal incentives that heightened hospitals’ awareness of cost control and efficiency [30].

Reducing the burden on patients has always been an important goal of healthcare reform in China [31]. We found that, during the first period of DRG reform, both patient cost-sharing and the patient sharing ratio showed a significant decreasing trend, which is consistent with research findings in China [32,33]. Notably, we did not observe an increase in the patient sharing ratio, as total hospital costs and medical insurance fund expenditures declined more rapidly than patient cost-sharing. Of course, the substantial cost reduction was likely driven by the synergistic effects of other policies accompanying the DRG reform [34], such as the zero-markup policy for drugs and medical supplies.

We observed that during the second period of the DRG reform, there were no significant monthly changes in cost and efficiency indicators, although these indicators increased compared to the first period. We speculate that two factors may explain this: On the one hand, hospitals progressively adapted to the DRG payment system, transitioning from an initial focus on cost control to prioritizing the core goal of standardizing medical practices. As a result, the control of costs and efficiency under the DRG system may have stabilized, a conclusion supported by previous studies [35]. On the other hand, hospitals may have been concerned that excessive cost control would lead to a continuous reduction in DRG payment standards, prompting them to raise total hospital costs to mitigate this effect [36].

Our results indicate that while the introduction of DRG somewhat slowed the increase in 30-day readmission rates, the monthly change was not statistically significant, consistent with findings from France [37]. The impact of DRG on healthcare quality, particularly readmission rates, remains a contentious issue [11,33,34,38]. This suggests that changes in readmission rates may not be directly attributed to the implementation of a specific policy [39] but could instead be influenced by patients’ healthcare needs or socioeconomic status [37].

In the lag analysis, we found that the introduction of DRG resulted in an immediate reduction in cost following a lag of 6 months. This may be because hospitals need time to adapt to new medical practices [5]. As a new payment system, DRG necessitates systematic training for healthcare managers to enhance cost awareness. In the early period of the reform, this effect was primarily reflected in a slower rate of cost growth, a finding consistent with a study conducted in Beijing [40].

It is a matter of concern that DRG-based payment systems may introduce financial incentives that drive unintended hospital behaviors. We observed a reduction in the proportion of patients aged 65 and above and the most substantial monthly reductions in total hospital costs and length of stay during the first period of DRG implementation. Older adults tend to have higher rates of comorbidities and functional impairments, which typically lead to higher costs compared to other age groups, often exceeding the DRG payment standard. To address potential financial pressures on hospitals treating complex elderly patients, Meishan Healthcare Security Administration has introduced an additional payment policy to mitigate the unintended risks associated with DRG [41].

Besides, one potential concern is patient selection (“cream skimming”), where hospitals preferentially admit patients with lower severity conditions who require fewer resources but yield the same DRG reimbursement. Another common issue is upcoding, in which hospitals exaggerate patient severity by recording additional complications to qualify for higher DRG payment categories [42]. Additionally, hospitals might adopt early discharge strategies, reducing the length of stay to lower per-case costs, which could lead to an increase in readmission rates [11]. A further possibility is resource reallocation, where hospitals compensate for revenue losses by shifting patients to outpatient services, rehabilitation centers, or self-financed treatments [43]. While our study does not directly measure these strategic responses, further evidence is needed to verify the occurrence of unintended behaviors.

### Recommendations

As DRG increasingly becomes a significant component of global healthcare payment systems, it has promoted the standardization and transparency of payment models. However, there are still some challenges that cannot be overlooked. In light of these issues, we propose the following policy recommendations:

First, healthcare insurance regulatory agencies should strengthen supervision and establish a scientific quality monitoring system to prevent unintended behaviors.

Second, it is essential to regularly organize reviews of DRG payment standards by clinical experts, economists, and statisticians to ensure their rationality and fairness, while further improving the DRG exclusion mechanism.

Third, financial incentives are a key driver of unintended behaviors. Hospitals should refine their performance evaluation systems to guide physicians toward a correct treatment philosophy, avoiding interference from purely economic motives in medical decision-making.

Finally, the successful implementation of DRG requires close collaboration among relevant departments to jointly promote the long-term governance of DRG reform.

### Limitations

This study has several limitations. First, the introduction of DRG system was accompanied by numerous confounding factors, especially during the COVID-19 pandemic, which affected both patient healthcare utilization and hospital management. Second, due to the inability to obtain data from non-pilot hospitals, the ITS analysis in this study did not include a control group, which limits the accuracy of causal inferences. Finally, our analysis was confined to the pilot city of Meishan in Sichuan Province, lacking cross-sectional comparisons with other pilot regions, which may limit the generalizability of the findings. Despite these limitations, this study provides valuable evidence for countries or regions considering the implementation of DRG system and contributes to China’s ongoing efforts to expand DRG reforms nationwide by 2025.

## Conclusions

The introduction of DRG in the early period effectively reduced total hospital costs, patient cost-sharing, patient sharing ratio, and length of stay, but did not significantly improve the rising trend in 30-day readmission rates. As the reform continued, the impact of DRG on cost control and service efficiency stabilized. However, hospitals may have adopted certain unintended behaviors in response to DRG incentives, which require further research to fully validate. Our results remind policymakers to strengthen the regulation of clinical practices, improve the DRG payment system, and continuously monitor trends in healthcare quality.

## Supporting information

S1 Table Interrupted time series (ITS) analyses for lag changes in outcome variables before and after the DRG. (DOCX)

S2 Table Interrupted time series (ITS) analyses for changes in outcome variables before and after the DRG by age group. (DOCX)

S3 Table Interrupted time series (ITS) analyses for value-for-money estimation before and after the DRG. (DOCX)
