# Supplementary material for: The impact of diagnosis-related groups on medical costs, service efficiency, and healthcare quality in Meishan, China: An interrupted time series analysis
Source: PLoS One. 2025 May 22;20(5):e0325041. doi: 10.1371/journal.pone.0325041 (PMC12097618; doi:10.1371/journal.pone.0325041)
Supplement: S1 Table — (DOCX) [file pone.0325041.s001.docx]

**S1 Table. Interrupted time series (ITS) analyses for lag changes in outcome variables before and after the DRG**

| Outcome Variables | Before DRG reform | First period of DRG reform | | | Second period of DRG reform | | |
| --- | --- | --- | --- | --- | --- | --- | --- |
|  | Baseline monthly  slope change (β1)  Coefficient (95% CI) | Immediate change(β2)  Coefficient (95% CI) | Monthly change(β3)  Coefficient (95% CI) | | Immediate change(β2)  Coefficient (95% CI) | Monthly change(β5)  Coefficient (95% CI) | |
| **Lag period: 1 month** | | | |  | | |  |
| Total hospital costs | 0.0033*** | 0.0125 | -0.0126*** | | 0.0694*** | 0.0036* | |
|  | (0.0014 - 0.0053) | (-0.0263 - 0.0514) | (-0.0164 - -0.0089) | | (0.0229 - 0.1159) | (-0.0002 - 0.0075) | |
| Patient cost-sharing | 0.0024** | 0.0336* | -0.0154*** | | 0.0916*** | 0.0025 | |
|  | (0.0004 - 0.0043) | (-0.0060 - 0.0732) | (-0.0192 - -0.0117) | | (0.0442 - 0.1390) | (-0.0014 - 0.0064) | |
| Patient sharing ratio | -0.0009** | 0.0209** | -0.0028*** | | 0.0219* | -0.0011 | |
|  | (-0.0019 - -0.0000) | (0.0026 - 0.0393) | (-0.0046 - -0.0011) | | (-0.0001 - 0.0439) | (-0.0029 - 0.0007) | |
| Length of stay | -0.0001 | -0.0129 | -0.0050*** | | 0.0334* | 0.0025 | |
|  | (-0.0017 - 0.0014) | (-0.0437 - 0.0179) | (-0.0080 - -0.0021) | | (-0.0035 - 0.0703) | (-0.0006 - 0.0055) | |
| 30-day readmission rate | 0.0085*** | -0.0670** | -0.0012 | | -0.0577 | -0.0022 | |
|  | (0.0052 - 0.0119) | (-0.1335 - -0.0005) | (-0.0076 - 0.0052) | | (-0.1366 - 0.0213) | (-0.0087 - 0.0044) | |
| **Lag period: 3 month** | | | |  | | |  |
| Total hospital costs | 0.0037*** | 0.0033 | -0.0154*** | | 0.0880*** | 0.0046** | |
|  | (0.0017 - 0.0056) | (-0.0311 - 0.0377) | (-0.0199 - -0.0110) | | (0.0406 - 0.1355) | (0.0007 - 0.0085) | |
| Patient cost-sharing | 0.0035*** | 0.0085 | -0.0202*** | | 0.1175*** | 0.0038** | |
|  | (0.0017 - 0.0053) | (-0.0237 - 0.0408) | (-0.0244 - -0.0160) | | (0.0730 - 0.1619) | (0.0002 - 0.0075) | |
| Patient sharing ratio | -0.0001 | 0.0054 | -0.0048*** | | 0.0294*** | -0.0008 | |
|  | (-0.0010 - 0.0007) | (-0.0104 - 0.0211) | (-0.0068 - -0.0028) | | (0.0076 - 0.0511) | (-0.0026 - 0.0010) | |
| Length of stay | -0.0003 | -0.0065 | -0.0059*** | | 0.0434** | 0.0030* | |
|  | (-0.0019 - 0.0013) | (-0.0346 - 0.0215) | (-0.0095 - -0.0023) | | (0.0046 - 0.0821) | (-0.0002 - 0.0062) | |
| 30-day readmission rate | 0.0076*** | -0.0214 | -0.0023 | | -0.0399 | -0.0016 | |
|  | (0.0040 - 0.0111) | (-0.0837 - 0.0408) | (-0.0103 - 0.0058) | | (-0.1251 - 0.0452) | (-0.0086 - 0.0055) | |
| **Lag period: 6 month** | | | |  | | |  |
| Total hospital costs | 0.0025** | -0.0161 | -0.0146*** | | 0.0889*** | 0.0049** | |
|  | (0.0001 - 0.0049) | (-0.0529 - 0.0208) | (-0.0194 - -0.0098) | | (0.0377 - 0.1401) | (0.0007 - 0.0092) | |
| Patient cost-sharing | 0.0026** | -0.0303* | -0.0191*** | | 0.1153*** | 0.0039* | |
|  | (0.0003 - 0.0048) | (-0.0651 - 0.0044) | (-0.0236 - -0.0146) | | (0.0670 - 0.1636) | (-0.0001 - 0.0079) | |
| Patient sharing ratio | 0.0001 | -0.0139* | -0.0045*** | | 0.0264** | -0.0010 | |
|  | (-0.0010 - 0.0011) | (-0.0304 - 0.0025) | (-0.0067 - -0.0024) | | (0.0035 - 0.0493) | (-0.0029 - 0.0009) | |
| Length of stay | -0.0013 | -0.0076 | -0.0051** | | 0.0425** | 0.0030* | |
|  | (-0.0032 - 0.0006) | (-0.0371 - 0.0220) | (-0.0089 - -0.0012) | | (0.0013 - 0.0837) | (-0.0005 - 0.0064) | |
| 30-day readmission rate | 0.0072*** | -0.0239 | -0.0011 | | -0.0413 | -0.0019 | |
|  | (0.0030 - 0.0114) | (-0.0886 - 0.0407) | (-0.0094 - 0.0073) | | (-0.1304 - 0.0477) | (-0.0094 - 0.0056) | |
| **Lag period: 12 month** |  |  |  | |  |  | |
| Total hospital costs | -0.0018 | -0.0060 | -0.0115*** | | 0.0919** | 0.0059* | |
|  | (-0.0058 - 0.0022) | (-0.0741 - 0.0621) | (-0.0182 - -0.0049) | | (0.0208 - 0.1629) | (-0.0007 - 0.0126) | |
| Patient cost-sharing | -0.0028 | -0.0444 | -0.0130*** | | 0.1067*** | 0.0030 | |
|  | (-0.0069 - 0.0014) | (-0.1140 - 0.0253) | (-0.0199 - -0.0062) | | (0.0339 - 0.1795) | (-0.0038 - 0.0098) | |
| Patient sharing ratio | -0.0010 | -0.0373*** | -0.0016 | | 0.0152 | -0.0028** | |
|  | (-0.0026 - 0.0007) | (-0.0653 --0.0094) | (-0.0043 - 0.0012) | | (-0.0142 - 0.0445) | (-0.0056 - -0.0001) | |
| Length of stay | -0.0043*** | 0.0052 | -0.0042* | | 0.0583** | 0.0037 | |
|  | (-0.0072 - -0.0014) | (-0.0434 - 0.0539) | (-0.0090 - 0.0005) | | (0.0073 - 0.1092) | (-0.0011 - 0.0084) | |
| 30-day readmission rate | 0.0031 | -0.0707 | 0.0029 | | -0.0219 | -0.0066 | |
|  | (-0.0025 - 0.0087) | (-0.1656 - 0.0241) | (-0.0064 - 0.0122) | | (-0.1204 - 0.0765) | (-0.0159 - 0.0028) | |

DRG denoted the Diagnosis-Related-Group; CI the confidence interval. All estimated coefficients were generated from generalized linear regression with log link. The estimated coefficients were directly interpreted as marginal effects. For example, an estimated coefficient of 0.0033 implied a 0.33% monthly increase in total hospital costs before the DRG reform. ITS analyses controlled for gender, age, insurance type, Charlson Comorbidity Index , hospital level, hospital ownership and seasonality.*p＜0.1；**p＜0.05；***p＜0.01
