# Supplementary material for: The impact of diagnosis-related groups on medical costs, service efficiency, and healthcare quality in Meishan, China: An interrupted time series analysis
Source: PLoS One. 2025 May 22;20(5):e0325041. doi: 10.1371/journal.pone.0325041 (PMC12097618; doi:10.1371/journal.pone.0325041)
Supplement: S3 Table — (DOCX) [file pone.0325041.s003.docx]

**S3 Table. Interrupted time series (ITS) analyses for value-for-money estimation before and after the DRG**

| Outcome Variables | Before DRG reform | First period of DRG reform | | Second period of DRG reform | |
| --- | --- | --- | --- | --- | --- |
|  | Baseline monthly  slope change (β1)  Coefficient (95% CI) | Immediate change(β2)  Coefficient (95% CI) | Monthly change(β3)  Coefficient (95% CI) | Immediate change(β2)  Coefficient (95% CI) | Monthly change(β5)  Coefficient (95% CI) |
| Total hospital costs | 30.41*** | -64.86 | -85.18*** | 359.64** | 26.08** |
|  | (18.42 - 42.40) | (-328.94 - 199.22) | (-109.73 - -60.63) | (76.87 - 642.40) | (1.52 - 50.64) |
| Patient cost-sharing | 14.80*** | -13.47 | -49.74*** | 187.33** | 9.36 |
|  | (8.40 - 21.20) | (-154.43 - 127.50) | (-62.85 - -36.64) | (36.39 - 338.27) | (-3.74 - 22.47) |
| Patient sharing ratio | -0.06*** | -0.14 | -0.08** | -0.71* | 0.12*** |
|  | (-0.09 - -0.02) | (-0.87 - 0.59) | (-0.15 - -0.01) | (-1.50 - 0.07) | (0.06 - 0.19) |
| Length of stay | 0.00 | -0.25** | -0.05*** | 0.27** | 0.02 |
|  | (-0.01 - 0.02) | (-0.49 - -0.00) | (-0.07 - -0.03) | (0.01 - 0.52) | (-0.01 - 0.04) |

DRG denoted the Diagnosis-Related-Group; CI the confidence interval. ITS analyses controlled for gender, age, insurance type, Charlson Comorbidity Index , hospital level, hospital ownership and seasonality

*p＜0.1；**p＜0.05；***p＜0.01
